# First diagnosis of familial partial lipodystrophy syndrome type 3 during pregnancy associated with a novel heterozygous *PPARG* variant and a concurrent *ABCC8* variant: a case report

**DOI:** 10.3389/fendo.2026.1855679

**Published:** 2026-06-22

**Authors:** Andreas Holstein, Ingy Jabri, Jonas A. Linck, Anke Tönjes, David J. F. Holstein, Peter Kovacs, Luise Pirlich

**Affiliations:** 1 Department of Gastroenterology, University Hospital for Ostwestfalen-Lippe, Klinikum Lippe, Detmold, Germany; 2 Department of Gynaecology and Obstetrics, University Hospital for Ostwestfalen-Lippe, Klinikum Lippe, Detmold, Germany; 3 Department of Urology and Paediatric Urology, Christliches Klinikum Paderborn, Paderborn, Germany; 4 Department of Medicine III, Division of Endocrinology, Nephrology and Rheumatology, University Hospital Leipzig, Leipzig, Germany; 5 Leipzig Center of Metabolism (LeiCeM), Leipzig University, Leipzig, Germany; 6 Department of Vascular and Endovascular Surgery, Evangelisches Diakonissenkrankenhaus Leipzig, Leipzig, Germany

**Keywords:** acute pancreatitis, FPLD3, hypertriglyceridemia, PPARG mutation, pregnancy

## Abstract

Familial partial lipodystrophy (FPLD) is a rare genetic syndrome characterised by persistent, selective loss of adipose tissue and is closely associated with severe metabolic disturbances. Pregnancy in women with FPLD is associated with a high risk for both mother and foetus, while clinical experience remains very limited. Evidence from case reports and small series is essential for risk stratification, multidisciplinary management, and optimization of maternal and foetal health. Here, we report the case of a woman at 18 weeks of gestation with very severe hypertriglyceridaemia complicated by acute pancreatitis. The patient had a history of young-onset diabetes mellitus, hypertension, polycystic ovary syndrome (PCOS) and previously known hypertriglyceridaemia, accompanied by characteristic loss of gluteofemoral and lower limb adipose tissue, raising the clinical suspicion of FPLD. Molecular genetic analysis was performed using next-generation sequencing-based gene panel diagnostics. Variants were described according to Human Genome Variation Society (HGVS) nomenclature and classified according to the American College of Medical Genetics and genomics/Association for Molecular Pathology (ACMG/AMP) guidelines. We made the initial diagnosis of severe FPLD3 syndrome and detected a novel heterozygous c.380A>C, p.(Glu127Ala) variant in the peroxisome proliferator-activated receptor gamma gene (*PPARG*), classified as likely pathogenic and considered causative for the patient´s phenotype. In addition, a heterozygous variant of uncertain significance c.328G>A, p.(Ala110Thr) in the ATP-binding cassette transporter sub-family C member 8 gene (*ABCC8*), was detected. High-dose intensive insulin therapy in combination with metformin and omega-3 fatty acids resulted in a marked reduction in triglyceride levels, normalised blood glucose levels until delivery and avoided further episodes of pancreatitis. Early recognition of FPLD is crucial, particularly in high-risk settings such as pregnancy. Intensive, multidisciplinary metabolic management can stabilize severe metabolic complications and may enable favourable maternal and foetal outcomes.

## 1 Introduction

Familial partial lipodystrophy (FPLD) is a rare genetic syndrome characterised by the persistent selective loss of adipose tissue and is closely linked to a variety of metabolic disturbances. These metabolic disorders include insulin resistance, diabetes, dyslipidaemia, severe hypertriglyceridaemia, low leptin and adiponectin levels, and metabolic dysfunction-associated steatotic liver disease (MASLD) (1). Consequently, FPLD often leads to early cardiovascular complications, as well as recurrent episodes of pancreatitis which significantly decrease the average life span. FPLD is a heterogeneous group of disorders that currently includes 11 recognized subtypes according to recent classifications (2). Among these, FPLD3 represents one of the most common subtypes (3). FPLD3 exhibits an autosomal dominant inheritance pattern and is based on loss-of-function mutations in the peroxisome proliferator-activated receptor gamma gene (*PPARG*) on chromosome 3p25.2 (1). PPARγ nuclear receptor is considered a key regulator of adipocyte differentiation, distribution and function, and mediates glyceroneogenesis, lipolysis, and the uptake, synthesis and storage of lipids. Individuals with FPLD3 exhibit a core phenotype characterised by loss of subcutaneous fat, particularly in the gluteofemoral region and distal upper limbs, as well as early-onset arterial hypertension and metabolic abnormalities. The extent of fat loss often determines the severity of metabolic derangements. The rarity of FPLD, together with its pronounced phenotypic heterogeneity and complex clinical manifestations, often leads to delayed diagnosis or misclassification. Pregnancy in women with FPLD3 is extremely rare and poses a high risk to both mother and child, presenting a challenge to all members of the treatment team. Our work presents the case of a complicated pregnancy in a woman with severe FPLD3 syndrome with a novel, likely pathogenic heterozygous variant c.380A>C, p.(Glu127Ala) in *PPARG*. In addition, we identified a further heterozygous variant of unclear significance c.328G>A, p.(Ala110Thr) in the ATP-binding cassette transporter sub-family C member 8 gene (*ABCC8*).

## 2 Case description

A 36-year-old primigravida at 18th weeks of gestation was admitted with acute epigastric pain, vomiting and nausea. Apart from insulin, she was not taking any other long-term medication. Physical examination revealed epigastric pressure pain. She was overweight (170 cm, 82 kg, BMI 28.4 kg/m²) and showed an abdominal fat distribution pattern. Notable features included hirsutism accompanied by diffuse alopecia, accentuated at the front and back of the head. Severe subcutaneous fat loss was identified at the buttocks, thighs and lower legs. There were no signs of acanthosis nigricans on the neck, armpits or groin. Eruptive xanthomas were absent.

### 2.1 Personal medical history

The refugee from Syria was not related to her husband. She experienced her first episode of pancreatitis at the age of 25. Concurrently, she was diagnosed with marked hypertriglyceridaemia, diabetes and arterial hypertension. Further evaluation for infertility led to the diagnosis of polycystic ovary syndrome (PCOS). Over the following years, she experienced six further episodes of pancreatitis. For the first time, she was prescribed insulin therapy and lipid-lowering drugs. Over the years, her diabetes therapy consisted of insulin and metformin. Since migrating to Germany four years ago, insulin has been discontinued in favour of various combinations of oral antidiabetic drugs (metformin, dipeptidyl peptidase IV inhibitors, dapagliflozin and repaglinide), semaglutide, statins with ezetimibe and omega-3 fatty acids. However, when she became unexpectedly pregnant in 2024, all oral antidiabetic drugs and lipid-lowering drugs had to be discontinued, and she was only treated with high-dose insulin therapy. Her antihypertensive treatment consisted of nifedipine.

### 2.2 Family history

Parents: The patient’s father died from an undiagnosed nervous system disease at the age of 73. Her mother suffered from long-standing diabetes and severe dyslipidaemia; she died of a myocardial infarction at the age of 62.

Siblings: The childless 54-year-old elder sister suffers from long-term diabetes, arterial hypertension and dyslipidaemia. She has been receiving dialysis treatment for four years. Her other elder sister (47 years old) also has diabetes and severe dyslipidaemia and recurrent episodes of pancreatitis. One of her two daughters died from pancreatitis at the age of 16, whereas her other daughter and her two sons are currently healthy. One of her six brothers died from alcoholic liver cirrhosis. Another brother suffers from coronary heart disease and has undergone coronary stenting, while the remaining four brothers are healthy. Each of the brothers has six healthy children.

## 3 Clinical timeline of the episode of care

**Table 1** shows the clinical timeline summarizing key events, metabolic findings and treatments from initial diagnosis to pregnancy, delivery and one year follow-up, including maternal metabolic course and neonatal outcome.

**Table 1:** Clinical timeline of disease course and management.

Time point/age	Gestational age	Key clinical events & findings	Treatment
Age 25	–	First episode of acute pancreatitis; diagnosis of hypertriglyceridaemia, diabetes mellitus, arterial hypertension, PCOS	–
Age 27	–	Six recurrent episodes of acute pancreatitis	First initiation of insulin and lipid-lowering therapy
Following years	-	Persistent diabetes mellitus and dyslipidaemia	Insulin + metformin
Age 32	-	Stable chronic metabolic disease (after migration to Germany)	Oral antidiabetic drugs; statin; omega-3 fatty acids
Age 36	18 weeks	Unplanned pregnancy; admission with hypertriglyceridaemia-induced acute pancreatitis and suboptimal gylcemic control	Intensive Care Unit, discontinuation of all oral drugs; intravenous fluids, heparin; continuous intravenous insulin
Hospital day 3-4	18 + 3-4 weeks	Rapid metabolic improvement	Switch to subcutaneous intensive insulin therapy
Remainder of pregnancy	19-36 weeks	Stable glycaemic and lipid control	high-dose insulin, add-on metformin; omega-3 fatty acids
	36 weeks	Delivery planning	Induction of labour followed by caesarean section
Delivery	-	Healthy male newborn (Apgar 9/9/10; 3, 250 g; 50 cm)	Neonatal monitoring
10 weeks postpartum	-	Severe immunodeficiency confirmed	Stem cell transplantation
age 1 year (child)	–	Child small for age; clinically stable	Ongoing follow- up

## 4 Diagnostic assessment, therapeutic intervention, follow-up and outcomes

**Table 2** shows the relevant laboratory investigations presenting extreme hypertriglyceridaemia and hypercholesterolemia. Leukocytes and other inflammation biomarkers were normal, as were liver function tests. Abdominal ultrasound revealed a fatty liver, but gallstones and extrahepatic cholestasis were excluded. In addition, serum lipase was elevated to 241 U/l, further supporting pancreatic involvement. The diagnosis of acute pancreatitis due to extreme hypertriglyceridaemia and suboptimal glycaemic control (HbA1c 9.6%) was made. The patient was referred to the intensive care unit (ICU). Haemodynamic status, electrolyte levels and urinary output were monitored, and intravenous fluid replacement consisted of 4, 000 ml of crystalloid infusion daily. In addition, heparin was administered subcutaneously. Regular insulin (4–6 IU/h) was administered intravenously to maintain blood glucose concentrations of 80-140 mg/dL. The patient remained in the ICU for four days. Within three days, triglyceride and cholesterol levels decreased from 9.621 mg/dL and 986 mg/dL initially to 2, 060 mg/dL and 545 mg/dL, respectively. The abdominal pain improved during the clinical course. Molecular genetic analysis was performed using next-generation sequencing (NGS) based on targeted gene panel diagnostics. Coding regions and flanking intronic sequences of clinically relevant genes were enriched and sequenced with high coverage, allowing reliable detection of single nucleotide variants and small insertions/deletions. Variants were annotated to current American College of Medical Genetics and Genomics/Association for Molecular Pathology (ACMG/AMP) guidelines, incorporating population frequency data (Genome Aggregation Database), in silico prediction tools and curated databases including Clinical Variation database (ClinVar) and Human Gene Mutation Database (HGMD).

**Table 2:** Most relevant laboratory findings at hospital admission.

Parameter (unit)	Finding	Reference
Blood glucose (mg/dl)	248	70-115
Lipase (U/l)	241	<60
C-reactive protein (mg/dl)	1.6	<0.5
Procalcitonin (ng/ml)	0.01	<0.02
Leukocytes (/nl)	8.3	4-9
Haemoglobin (g/dl)	11.8	14-18
Hematocrit (%)	31.6	42-52
Triglycerides (mg/dl)	9621	<150
Cholesterol (mg/dl)	986	<200
HDL (mg/dl)	<3	>40
LDL (mg/dl)	n.a.	<160
Apolipoprotein(a) (mg/dl)	<5	<30
Apolipoprotein(b) (mg/dl)	138	55-130
Haemoglobin A1c (%) Haemoglobin A1c (mmol/molHb)	9.6 83	<6.5 20-42
Fasting C-peptide (µg/l)	3.6	0.8-4.2
Anti-glutamic acid decarboxylase antibodies (IE/ml)	1.0	<10
Islet cell antibodies (U/ml)	0.1	<0.4
Zinc transporter 8 antibodies	Neg.	Neg.
Insulin Antibodies	Neg.	Neg.
Creatinine (mg/dl)	0.72	0, 7-1.2
eGFR (ml/min)	110	>90
Bilirubin total (mg/dl)	0.3	<1.2
Gamma-GT (U/l)	21	<40
GOT (U/l)	22	10-35
GPT (U/l)	31	<35
TSH (µE/ml)	2.12	0.3-3.5

Molecular genetic testing revealed a novel, likely pathogenic heterozygous variant NM _138711.6(*PPARG*):c.380A>C (p.Glu127Ala) in *PPARG* (ClinVar database accession: SCV006083425.1), considered causative for the patient`s FPLD3 phenotype. Classification as likely pathogenic was based on the ACMG/AMP criteria Pathogenic Moderate 1 (PM1), Pathogenic Moderate 2 (PM2), Pathogenic Moderate 5 (PM5) and Pathogenic Supporting 3 (PP3). Specifically, the variant is located within the highly conserved DNA-binding domain of PPARγ, a critical functional region enriched for disease-causing variants (PM1), is absent or extremely rare in population databases (PM2), affects an amino acid residue at which a different pathogenic missense variant has previously been reported (PM5) and is predicted to have a deleterious effect by multiple in silico prediction tools (PP3).

In addition, a heterozygous variant NM_000352.6(*ABCC8*):c.328G>A (p.Ala110Thr) of uncertain significance in *ABCC8* (ClinVar database accession: Accession: SCV006083424.1) was detected. According to ACMG/AMP criteria, this variant fulfilled PM1 and PM2, reflecting its localization within a functionally relevant region of *ABCC8* and its absence or extreme rarity in population databases. However, the available evidence was insufficient for classification as pathogenic or likely pathogenic and the variant was therefore classified as a variant of uncertain significance.

Until delivery, high-dose intensive insulin therapy (subcutaneous(s.c.) >140 IU insulin lispro and degludec daily) with additional metformin (maximum 2x1000 mg/day) normalised blood glucose levels (HbA1c 6.1%). Further therapy involving omega-3 fatty acids maintained triglyceride levels between 1100 and 1500 mg/dL and cholesterol levels between 206 and 290 mg/dL.

### 4.1 Course of pregnancy and foetal and maternal outcome

Following careful interdisciplinary consultation, induction of labour by amniotomy and infusion of oxytocin was performed in the 36th week of pregnancy. Following a suspicious cardiotocogram, maternal exhaustion and a suspected intrauterine infection, an uncomplicated caesarean section was finally performed. The newborn boy was healthy (Apgar scores of 9/9/10; weight 3, 250 g; length 50 cm) and unremarkable. Following treatment for prolonged hyperbilirubinaemia, the mother and child were discharged after two weeks.

Shortly thereafter, the infant developed recurrent infections and palmar and plantar erythema, as well as experiencing poor physical development. Based on specific laboratory findings and genetic testing, a diagnosis of atypical severe combined immunodeficiency as part of cartilage-hair hypoplasia, with a highly reduced number of T cells (CD4+ and CD8+), was made. Ten weeks after his birth, the boy underwent successful allogeneic stem cell transplantation (fresh bone marrow). Two probable pathogenic variants in the RMRP (RNA component of mitochondrial RNA processing endoribonuclease) gene were detected. Currently, at the age of 18 months, the boy is small for his age but in good condition.

Regarding maternal follow-up, the patient is currently in stable condition. Her height is 170 cm. Pre-pregnancy body weight was 75 kg (Body-Mass-Index: 26, 0 kg/m²), increased to 85 kg during pregnancy and returned to 75 kg postpartum. Current antidiabetic treatment consists of metformin, dapagliflozin, repaglinide and semaglutide. Antihypertensive treatment includes ramipril and lipid lowering therapy consists of ezetimibe/atorvastatin, fenofibrate and omega-3 fatty acids. Further evaluation regarding potential leptin-based therapy is planned. How ever follow-up has been challenging due to limited contact with the patient. Comprehensive phenotypic characterization by formal body composition assessment was not performed during pregnancy or postpartum. In addition, clinical photographic documentation was not available because consent was not granted by the patient. This should be considered a limitation of the present report.

## 5 Discussion

Our 36-year-old patient showed the characteristic phenotypic features of partial lipodystrophy syndrome. The typical metabolic disturbances included diabetes, insulin resistance, and severe hypertriglyceridaemia and hypercholesterolaemia, which led to repeated episodes of pancreatitis. Type 1 diabetes could be ruled out, as specific antibodies were negative. Significant residual beta-cell function, as indicated by a high C-peptide level, suggested insulin resistance. Furthermore, fatty liver disease and reproductive disorders, including PCOS, were verified.

Patients with familial partial lipodystrophy represent a high-risk cardiometabolic population with markedly increased long-term morbidity and premature mortality. The combination of severe insulin resistance, atherogenic dyslipidaemia, chronic hyperglycaemia, hepatic steatosis and hypertension promotes accelerated atherosclerosis and end-organ damage, often manifesting at a young age. In addition to recurrent pancreatitis, affected individuals show an increased risk for cardiovascular disease, progressive liver disease and microvascular diabetic complications (4). Consequently, early recognition of FPLD is essential, as management requires a strict, multimodal therapeutic strategy targeting all metabolic components simultaneously. Intensive glycaemic control, aggressive lipid lowering, blood pressure optimization and lifestyle interventions are critical to mitigate long-term cardiovascular risk. Treatment must be individualized and closely monitored, particularly in situations such as pregnancy where therapeutic options are limited (4). Outside pregnancy, targeted therapies addressing adipose dysfunction may complement conventional diabetes treatment. In *PPARG*-related lipodystrophy (FPLD3), thiazolidinediones may be of particular interest due to their *PPARG*-mediated effects on adipocyte differentiation and insulin sensitivity, although therapeutic responses may depend on the underlying genotype. In the present case, thiazolidinediones were not used. GLP-1 receptor agonists may reduce ectopic lipid burden, while leptin replacement represents a disease-specific approach with metabolic benefit in lipdodystrophy (5).

A definitive diagnosis of FPLD3 in the described case was made by identifying the novel c.380A>C (p.Glu127Ala) variant in the *PPARG* gene. There is a well-documented set of other rare missense variants in *PPARG* that reduce receptor/transcription-factor function and are associated with metabolic disease (6). Loss-of-function and dominant-negative missense variants in *PPARG* are well-established causes of FPLD3 and severe insulin resistance/metabolic syndrome (6). Position 127 lies within the AF-1 (activation function-1) domain, which is the transcriptional activation domain that regulates coactivator interactions and can modulate transcription independently of ligand binding. Thus, p.Glu127Ala may reduce the transcriptional activity of PPARγ, potentially leading to a partial loss of function. Although there is a lack of direct experimental data for the single E127A substitution, the double mutant C126A/E127A has been used and validated repeatedly as a DNA-binding-deficient (cis-activation null) PPARγ. These studies have shown that altering Glu127 (in combination with Cys126) eliminates DNA binding/transactivation (7). Overall, c.380A>C (p.Glu127Ala) represents a rare missense variant in *PPARG* with plausible functional relevance and was classified as novel, likely pathogenic according to ACMG criteria. Nevertheless, published functional and clinical evidence for this specific variant remains limited and further experimental studies are warranted to better characterize its pathogenic mechanisms.

Additionally, we detected a novel variant c.328G>A (p.Ala110Thr) of uncertain significance in the *ABCC8*. The *ABCC8* encodes SUR1, the regulatory subunit of the pancreatic β-cell ATP-sensitive potassium (KATP) channel. Variants in *ABCC8* can disrupt KATP-channel function, affecting insulin secretion (8). Ala110Thr lies in the N-terminal transmembrane region of SUR1, which interacts with Kir6.2 to regulate channel gating. Although functional studies for this exact variant are missing, most variants in this region cause loss-of-function, resulting in reduced KATP channel activity and excess membrane depolarization leading to congenital hyperinsulinism (6). However, some autosomal dominant gain-of-function *ABCC8* missense variants can lead to impaired insulin secretion and may result in Maturity-Onset Diabetes of the Young (MODY)/neonatal diabetes mellitus (NDM) (6). The clinical phenotype of our patient and her relatives suffering from diabetes would support the gain-of-function mechanism for the Ala110Thr variant. Without functional or segregation data, it would likely be classified as variant of unclear significance.

Overall, the *PPARG* variant is considered the most likely genetic cause of the patient’s FPLD3 phenotype. In contrast, the contribution of the *ABCC8* variant remains uncertain because it is currently classified as a variant of uncertain significance. Although a modifying effect on the metabolic phenotype cannot be excluded, this remains speculative and requires further investigation.

The identification of the two novel variants is consistent with the patient’s family history, which strongly suggests a genetic background of autosomal dominant inheritance. The mother of our patient, all her sisters and a niece who died from pancreatitis were affected by diabetes, dyslipidaemia, hypertension, episodes of pancreatitis and early death. Unfortunately, due to pragmatic reasons, genetic testing could not be conducted on the other living family members who are still in Syria. We also have no anthropometric data on the other family members.

Pregnancy is generally a rare condition in all types of FPLD. FPLD2 (Dunnigan type), which is caused by pathogenic variants in the *LMNA* gene, is associated with infertility rates of 30% and a prevalence of PCO exceeding 50%. Nevertheless, some pregnancies have been reported. In these small case series, there were high complication rates, including spontaneous abortions, diabetes, premature birth and pre-eclampsia, as well as malformations and neonatal deaths. Pregnancy in FPLD3 remains extremely rare. PPARγ is considered to play an essential role in placenta formation, which is crucial for embryo implantation, maintaining pregnancy, and foetal development and growth. Therefore, the presence of functional PPARγ protein appears essential for placental angiogenesis and foetal growth. To our knowledge, our triglyceride and cholesterol levels of nearly 10, 000 mg/dl and 1, 000 mg/dl, respectively, are the highest so far reported in FPLD3 associated with pregnancy. The treatment of severe dyslipidaemia during pregnancy and breastfeeding remains challenging because therapeutic options are restricted and evidence supporting the use of most lipid-lowering agents in these settings is limited. Therefore, management requires an individualized approach balancing maternal metabolic control against potential foetal and neonatal considerations. In our patient, intensive insulin therapy combined with metformin resulted in a rapid and marked reduction in triglyceride and cholesterol levels within three days of ICU admission. In addition, omega-3 fatty acids were administered as part of the lipid-lowering strategy.

Under this treatment regime recurrence of pancreatitis could be avoided. Alternatively, the performance of lipid apheresis should have been considered (9).

**Table 3** summarizes the main genetic subtypes of familial partial lipodystrophy and their pregnancy-related manifestations, including the underlying pathophysiology, clinical metabolic course and obstetric outcomes. Across subtypes, pregnancy is frequently complicated by worsening insulin resistance, dyslipidaemia and hypertensive disorders, with *PPARG*-related FPLD showing the severest metabolic deterioration and neonatal complications, whereas *LMNA* and *PLIN1* variants demonstrate a heterogeneous but generally more favourable outcome when adequate metabolic control is achieved.

**Table 3:** Genetic subtypes of familial partial lipodystrophy (FPLD) and pregnancy-related manifestations reported in the literature.

FPLD subtype	Gene, mutations & references	Pathogenetic mechanism	Clinical features during pregnancy	Pregnancy outcome
FPLD3	***PPARG*** • (10) • (9) • (11) • Own case: c.380>C, p (Glu127Ala) ***ABCC8*** • Own case: c.328G>A, p.(Ala110Thr)	Impaired PPARγ transcriptional activity (dominant-negative or loss-of-function) → defective adipocyte differentiation, severe insulin resistance, dyslipidaemia; role in placental development	• **Castell et al., 2012:** High dose insulin (200 IU), diabetes well controlled (HbA1c <6%)	Delivery at 31 week; hydrops fetalis with death within 24 hours
			• **Madhra et al., 2012:** Severe hyperglycaemia and hypertri- glyceridaemia; plasmapheresis, high-dose insulin; poor compliance	Caesarean section at 32 week; child with developmental delay
			• **Gosseaume et al., 2023:** 6/25 pregnancies did not result in birth; preterm delivery (<37 week)	Premature delivery (<37 week); lower birth weight in dymetabolic pregnancies
			• **Own case:** High dose insulin+ metformin; well controlled diabetes (HbA1c 6.1%), Omega- 3 fatty acids administered; Triglycerides 1100-1500 mg/dl; cholesterol 206-290 mg/dl	Caesarean section at 36 week; neonatal course: prolonged hyperbilirubinaemia and severe combined immunodeficiency requiring stem cell transplantation (10 weeks)
FPLD2	***LMNA*** • (12): p.Arg482Trp • (13): p.Arg482Gln	Disruption of nuclear lamina → impaired adipocyte differentiation and metabolic dysregulation	• **Vantyghem et al., 2008** Gestational diabetes mellitus; increased insulin resistance; hypertension/preeclampsia; higher miscarriage and adverse outcomes	Increased miscarriage, complication and fetal deaths; overall elevated risk of adverse pregnancy outcomes
			• **Valerio et al., 2024** Gestational diabetes mellitus (subset) metabolic deterioration; hypertension in some cases; heterogeneous maternal and fetal outcomes	Mostly live births; no consistent malformations; occasional preterm delivery; generally favorable neonatal outcomes
FPLD4	***PLIN1*** *• * ***(***14) heterozygous *PLIN1* mutations	Defective lipid-droplet formation and triglyceride storage	• **Frolkova et al., 2025** Insulin resistance; dyslipidaemia and high-risk metabolic profile due to lipodystrophy	Successful pregnancy; live birth of a healthy neonate; no major complications

Bold and italic values indicate gene names. Bold values indicate references from the literature.

Apart from prolonged neonatal hyperbilirubinaemia, our newborn’s condition was initially unremarkable. However, 10 weeks after birth, the child developed atypical severe combined immunodeficiency (SCID) as part of cartilage-hair hypoplasia. This necessitated allogeneic stem cell transplantation. SCIDs are a group of inborn errors of the immune system that are usually associated with severe or life-threatening infections. We assume that there are no significant links between FPLD and the risk of SCID in the offspring of women with FPLD. Nevertheless, this coincidence is notable.

## 6 Patient perspective

Arabic (native language):

ع ن د تشخيصي بالحالة خلال فترة الحمل، كان ت التجربة في البد اية صاد مة بالن سبة لي. ومع ذلك، وجد ت أن الخطة الع لاجية المقد مة كان ت مقبولة، ولم أع ان ِ من أي آثار جان بية ن تيجة الع لاج. لم يؤثر المرض أو الع لاج ع لى حياتي اليومية أو جود ة حياتي أثن اء الحمل، حيث سارت الأمور بشكل طبيع ي. ع لى الصع يد الن فسي، شع رت ببع ض الخوف والقلق، خاصة فيما يتع لق بصحة الجن ين ، إلا أن ن ي تلقيت د ع مًا كبيرًا من الفريق الطبي، وشع رت بالتفهم ووضوح المع لومات المقد مة في جميع مراحل المتابع ة، وأن ا ممتن ة لهم جميع ًا. بع د الولاد ة، أن ظر إلى التجربة بشكل إيجابي، ولا يوجد لد ي أي قلق حالي أو مخاوف مستقبلية متع لقة بالحالة.

English translation:

Being diagnosed with this condition during pregnancy was initially a shock for me. However, I found the proposed treatment to be acceptable and did not experience any side effects related to the therapy. The condition and its management did not negatively affect my daily life or quality of life during pregnancy, and everything progressed normally. From a psychological aspect I did experience some fear and concern, particularly regarding the health of my baby. Throughout the process I felt well supported by the medical team and appreciated their understanding and the clarity of the information provided. I am grateful to all of them. After delivery I view the experience positively and do not have any ongoing concerns or worries related to my condition.

## Data Availability

The datasets presented in this study can be found in online repositories. The names of the repository/repositories and accession number(s) can be found in the article/supplementary material.
